# Total pain in the indirect victims of the *desaparecidos*

**DOI:** 10.3389/fsoc.2026.1808511

**Published:** 2026-05-11

**Authors:** Gina Tarditi

**Affiliations:** School of Social and Environmental Sustainability, University of Glasgow, Dumfries, United Kingdom

**Keywords:** ambiguous loss, *desaparecidos*, disenfranchised grief, suffering, the disappeared, total pain

## Abstract

This article explores the phenomenon of the *desaparecidos—the disappeared,* mostly enforced—in Latin America, with a specific emphasis on Mexico. Little is known about the multidimensional impact that a disappearance has on the relatives of the *desaparecidos*. Government protocols supporting indirect (secondary) victims only partially address legal issues, provide limited economic assistance, and offer perfunctory responses to their psychosocial needs. From the author’s perspective, the concept of ‘total pain’ effectively captures the multi-layered suffering endured by the indirect victims. Framing the multidimensional suffering of indirect victims as ‘total pain’ can inform stronger care protocols and public health policies as they navigate the long-term effects of the disappearance.

## Introduction

Enforced disappearances^1^,^2^ have been a social and political issue in Mexico and other Latin American countries since the second half of the 20th century (Aureliani, 2023). Initially, disappearances were carried out by the military to control “subversive” groups (Aureliani, 2023), especially from the 1960s to the 1990s, during the “dirty war” period (López and Urbina Cortés, 2025). However, in Mexico, disappearances have steadily risen since 2007, when the president declared a war on drugs. Most disappearances in Mexico are enforced (Lomnitz, 2025), occurring either through direct action, acquiescence, or complicity of the state,^3^ and the term *desaparecidos* is commonly used in scholarly literature, among activists, and in the media to refer to the disappeared.

The General Law on Enforced Disappearance of Persons, Disappearance Committed by Individuals, and the National System for the Search of Persons^4^ was enacted in Mexico in 2017 to establish a coordinated national system to improve the search for disappeared persons and the identification of unidentified bodies, but has not been fully implemented (Human Rights Watch, 2025). Comisión Nacional de Búsqueda (2026), whose aim is to assist indirect victims, has been judged insufficient and ineffective (Amnesty International, 2025). A DNA database has not been established, even though there are more than 72,100 unidentified human remains (Amnesty International, 2025). The data from the official registry for enforced disappearances and missing persons (Registro Nacional de Personas Desaparecidas y no Localizadas, 2026) is not properly systematized (Gatti, 2022). Furthermore, on March 27th, 2026, the Mexican president presented a data reclassification, according to which, of a total of 132,534 cases, 33% have shown no activity since disappearance, 31% have some activity but remain untraceable, and the remaining 36% lack sufficient information to initiate a search (Sheinbaum, 2026). This announcement prompted an immediate reaction from several organizations, who interpret this as an attempt to disappear the *desaparecidos* (Data Cívica, 2026).

Disappearances in Mexico take many forms in contrast to other countries (Gatti, 2022). People disappear not only for political reasons; many are victims of slavery, trafficking, forced labor, gender violence, and abuse occurs by both criminal groups and military authorities (Rodríguez Fuentes, 2017). Approximately 75% of all *desaparecidos* in Mexico are men aged 15–40, although children and women are also disappeared (Aureliani, 2023). They include students, migrants, journalists, politicians, and human rights activists (Aureliani, 2023). Accordingly, the multicausality involved in this phenomenon requires a different approach to understanding and addressing the needs of indirect victims (Robledo Silvestre, 2015).

Regardless of cause, the families of the *desaparecidos* aspire to find their missing relatives, to learn the truth, and to achieve justice (Robledo Silvestre, 2015). The experience of indirect victims has been described as a “complex and unique experience” (Lenferink et al., 2019, p. 298), as one of the most dramatic challenges faced by Mexico (López and Urbina Cortés, 2025), and as a wound that often transcends families intergenerationally (Faúndez et al., 2017). Hence, this type of loss is distinct due to complex legal and political issues, a prolonged liminal state, and the significant disruption to the families’ narratives and biographies (Comité Internacional de la Cruz Roja, 2014). At the same time, indirect victims often endure the inaction, ineptitude, and corruption of the authorities, amid high indices of impunity—up to 98.8% (Arcos Robledo, 2025). As for psychological support, when it exists, there is no formal follow-up or individualized care plan (López Cruz and Romero Vargas, 2024).

## Secondary victims and searchers

More than 230 collective search movements have been organized by indirect victims in Mexico, mainly women, to challenge official narratives and exercise agency in searching for *desaparecidos*, demanding truth, and seeking justice (Amnesty International, 2025; Human Rights Watch, 2025). Search collectives are key to understanding the phenomenon. They create grieving communities, particularly in vulnerable socio-cultural areas across Mexico (Rodríguez Aguilera, 2022), and, united by their suffering, help one another in their search and give missing relatives an identity (Irazuzta, 2020). They protest, wearing t-shirts featuring images of *desaparecidos,* and repeating powerful slogans, such as “until we find them.” Using pick and shovel, they have dug in more than 5,000 clandestine graves where direct victims were buried (Human Rights Watch, 2025). The search has become their mission, and they often express their experience as being embodied:

I heard on the radio that when you lose someone, you have heartache, but that is not true; you get a stomach ache forever (Murillo, 2025, p. 54).

Physically, they experience insomnia, sleep problems, fatigue, and general deterioration in well-being (López Cruz and Romero Vargas, 2024). Those with pre-existing illnesses sometimes experience aggravation of their condition (Barrera Cortés and Veraza González, 2023). Emotionally, they experience continuing distress and may develop anxiety, depression, suicidal ideation, or substance abuse (Barrera Cortés and Veraza González, 2023). Socially, they often are estranged or excluded from their communities (Barrera Cortés and Veraza González, 2023). Hundreds have been forced to abandon their land due to violence and criminalization (López and Urbina Cortés, 2025). They face social stigma, as it is frequently assumed that their *desaparecido* was involved in criminal activity (Bourguignon et al., 2021). A mother, leader of a collective, reacted on X to the revictimization she suffered when she recognized that one of her three missing children had been involved with a criminal group,

[…] if someone committed a crime, they should be in prison, not dead or disappeared.^5^ (Flores, 2025)

Families, particularly mothers, live in a liminal space (Turner, 2018), never certain if their *desaparecidos* are alive or dead (Camacho Quirós, 2024). They face impoverishment and a lack of support to carry out parenting roles, and the search for their *desaparecidos* (Barrera Cortés and Veraza González, 2023). The entire family’s stability is affected including children, who sometimes exhibit mental and physical issues (Comité Internacional de la Cruz Roja, 2014; Hernández-Brussolo et al., 2022). They also put their safety at risk in suffering verbal or written threats, extortion, robbery, and torture. Since 2010, more than 27 searching victims have been killed (Nuño and Ayala Martínez, 2025). Furthermore, scholars have investigated how this painful and traumatic experience is often intergenerationally transmitted (Faúndez et al., 2017).

Existentially, secondary victims struggle to make sense of their experience and maintain hope (Comité Internacional de la Cruz Roja, 2014). This suffering deepens further due to the absence of a body to grieve, remains to dispose of, or rituals to perform (Boss, 2017; López and Urbina Cortés, 2025). During a meeting with authorities, the father of a *desaparecido,* Gustavo Hernández, broke the silence with this appeal: “at least give me a little bone to allow him a Christian burial”^6^ (El Financiero, 2025). His words reflect the complex suffering that the lack of a body poses for these families (López and Urbina Cortés, 2025). Overall, this crime causes serious harm on multiple levels: the victims (micro), the community (meso), and the entire society (macro) (Hernández-Brussolo et al., 2022).

The experience of the indirect victims of the *desaparecidos* has been examined through different theoretical approaches, such as ambiguous loss (Almanza et al., 2019); prolonged or complicated grief (Lenferink et al., 2019); suspended grief (Bekerman et al., 2009); post-traumatic stress disorder (Isuru et al., 2019); and psychoanalytical (Mejía and Aguirre, 2014). While each of these approaches has strengths, it has been argued that they fail to fully comprehend the victims’ experiences because they do not account for the complexities involved, including political, legal, and sociocultural aspects, resulting in unintentional pathologization (Hernández-Brussolo et al., 2022). However, overall, in relation to the phenomenon of disappearances, grief literature is scant (Wayland et al., 2016). The most common theoretical frameworks are those of two American psychologists; Pauline Boss’s concept of ambiguous loss (Boss, 1999), and Kenneth Doka’s concept of disenfranchised grief (Doka, 1989).

## Ambiguous loss and disenfranchised grief

The American psychologist Pauline Boss introduced the concept of ambiguous loss to describe “an unclear loss—a loved one missing either physically or psychologically” (Boss, 1991, p. 237). She differentiates two types of ambiguity: when the person is physically absent but psychologically present (e.g., *desaparecidos*), or physically present but psychologically absent. Boss has studied ambiguous loss in the context of *desaparecidos* in Latin America, and has called for understanding it as a complex, external stressor rather than a consequence of an individual’s weakness or pathological behavior (Boss, 2002). Boss makes a clear distinction between post-traumatic stress disorder and the trauma caused by the physical absence/psychological presence of a missing person. The main difference lies in the trauma’s temporality. In the first case, the traumatic event took place in the past, its aftermath is what we know as post-traumatic stress disorder. In the second case, the trauma is ongoing and may last for the rest of the victim’s life (Boss, 2017). The International Committee of the Red Cross (Comité Internacional de la Cruz Roja, 2014) and some Mexican scholars (Hernández-Brussolo et al., 2022) have highlighted these distinctive trauma characteristics. The model has also been used by others to describe experiences of grief in this context (e.g., Almanza et al., 2019; Wayland and Maple, 2020).

The American gerontologist Kenneth Doka introduced the concept of disenfranchised grief in 1989 to describe a response to a loss that is not recognized or validated by the social group. In the case of a *desaparecido*, social norms and the authorities frequently attribute blame to the direct victim for criminal conduct, or to the indirect victims for inadequate parenting (Amnesty International, 2025). Consequently, secondary victims lack validation for their loss, often facing indifference or incomprehension from their communities and the authorities, and struggle with multiple legal barriers (Bourguignon et al., 2021; Doka, 2025).

Together, ambiguous loss and disenfranchised grief are present in most cases of the *desaparecidos*’ indirect victims, and specialized literature asserts these are helpful therapeutic approaches for them (Wayland et al., 2016). However, I believe that neither ambiguous loss nor disenfranchised grief can fully articulate the embodied experience of suffering that these indirect victims of disappearance endure. Although this literature often addresses the lack of social and public recognition and the stigmatization that usually accompanies the victims’ experience, there remains a lack of theorizing the suffering of these victims, understood as a multifaceted experience crossing physical, emotional, social, and existential domains—not only at the individual but also collective levels. Consequently, I explore whether we could better understand the victims’ embodied suffering by considering it as ‘total pain’ (Saunders, 1964).

## Total pain

In 1964, Cicely Saunders introduced the concept of ‘total pain’, which is regarded as a central development in modern medicine, particularly in palliative medicine. This concept emerged within a specific historical “time of war, exile and displacement,” as well as post-war advances in how pain was understood (Wood, 2024, p. 68). Saunders sat with her terminally ill patients and listened to them describe the meanings their pain held for them personally. One of her most famous patients was Mrs. Hinson, who responded to Saunder’s question of how she was, saying, “well, doctor, the pain began in my back, but now it seems that all of me is wrong” (Saunders, 1964, p. viii), and then continued narrating her symptoms, her family’s difficulties, and her spiritual struggle to make any sense of what was happening. The pain that began by affecting the patient’s body had extended to her mind, spirit, family, finally invading her entire social and cultural world (Saunders, 1964). From this and many other patients’ narratives, Saunders advocated the impossibility of separating physical pain from that of the mind, the spirit, and social relations (Saunders, 1981).

Saunders, a woman with strong philosophical and deep spiritual roots, continued exploring the concept of “total pain” from her unique background as a nurse, social worker, and medical doctor (Wood, 2022). She realized that the families of her patients showed emotional distress and often struggled with unsolved familial issues (Saunders, 1981). The patients’ individual pain extended to their familial and social networks. Families also grappled with existential issues, anticipating the death of their relatives, searching for meaning and reconciliation in cases of past regrets. Furthermore, families often lacked privacy and appropriate environments in which to express their needs and be listened to Saunders (1981).

Saunders also noted that staff paid an emotional toll and struggled to balance time between supporting their patients and meeting their own personal needs (Saunders, 1981). Moreover, the phrase “there is nothing more to be done” was common at the time (Saunders, 1967, p. 162), leading many doctors to desert their patients at late stages of their disease, leaving them isolated and aggravating their suffering (Saunders, 2006). Thus, both the patients’ families and staff experienced emotional and existential suffering, expressed as confusion, a lack of meaning, regret, and guilt (Wood, 2022). Saunders concluded that patients and their families should be considered a unit to be cared for, and advocated for interdisciplinary work teams to meet the multidimensional needs of patients, their families, and staff (1981). After years of experience, she went on to claim that “total pain required total care” (Saunders, 1967, p. 163).

Although the concept of “total pain” originated in Saunders’ work with patients at the end of life, I believe it has the virtue of encapsulating the experiences of suffering in the aftermath of disappearances. First, Saunders described “total pain” not as a single event but as a situation that begins in the body and extends to the patient’s entire inner and social world (Wood, 2022). Similarly, in the case of the indirect victims of the *desaparecidos*, suffering is not a single event. As Boss (2002) describes, they inhabit a state of physical absence and psychological presence of their *desaparecidos*, a painful experience that shapes their emotional, social, spiritual and even physical worlds. Though the origins of pain differ, in both cases suffering extends to all the individual’s realms and can be palliated but not necessarily resolved, because the causes are irreparable.

Second, just as dying patients and their families were and still may be abandoned by the staff who are supposed to accompany them along the way, indirect victims of disappearance are frequently abandoned by the state and sometimes by their communities and social networks. This can be partly explained by the inability of care providers to engage in emotion work due to systemic constraints, as well as by the complexity of victims’ embodied experiences. As Wood puts it, “complex experiences of pain and suffering resist communication” (2021, p. 417). However, Saunders found that listening to patients’ narratives yielded important cues, in the form of metaphors, small bites of information, anecdotes, or even silences that, altogether, provided the necessary information to grasp better their unique ‘total pain’ experience (Saunders, 2006; Wood, 2022). Saunders demonstrated that in caring for those who suffer, both knowledge and presence are crucial. The act of listening can be therapeutic (Krawczyk, 2018), promoting open dialogue and, according to Saunders, even reduce the patient’s perception of pain (Wood, 2022). Saunders believed that it was possible to communicate these experiences to broader audiences to foster change and improve institutional and cultural responses to the care of the dying (Wood, 2022). Saunders’ approach helped with patients reclaim personal identity and larger representation in the world.

In relation to indirect victims, the question is whether we could also learn about their embodied experiences of suffering by actively listening to what they have to say and using that knowledge to foster better supports for them. Listening to the narratives of the indirect victims is especially relevant in the context of disappearances in Mexico, where silence from the authorities and society prevails, the facts are often denied, and the direct victims are frequently made invisible (Robledo Silvestre, 2015; Rodríguez and Herrera Bautista, 2025).

Third, the earliest origins of the concept of ‘total pain’ date back to the Second World War, when Saunders became a nurse. Later, in 1948, she established a relationship with her first patient, the Polish David Tasma (Wood, 2024, p. 67). Tasma was facing the end of his life, but he was also struggling with lasting wounds from his personal history of war and displacement (Wood, 2024). From this and other patients’ experiences, Saunders realized that the individual’s experience of pain was shaped not only by social, biographical, and existential elements, but also by their wider socioeconomic, political, and historical contexts (Wood, 2024). Saunders’ careful listening to her patients’ and families embodied and situated stories, not only of the physical pain accompanying their terminal illness, but also about war and displacement, albeit partial and fragmented, was an important enabler in her finding new ways to address “total pain.”

## Conclusion

The indirect victims of the *desaparecidos* in Mexico live with ambiguous and disenfranchised loss inflicted by violence. At the same time, I argue that “total pain” both builds on and extends multidimensional understanding of this suffering beyond these concepts. It does so by addressing multiple dimensions of suffering; treating suffering not as a discrete event but as a situation that necessarily includes those who are not seen as the “primary” victim; foregrounding structural and social abandonment and, with it, the importance of listening and accompaniment; recognizing the significance of biographical disruption; and situating suffering within the sociocultural, historical, and political contexts that shape how it is lived. Doing so provides a holistic, flexible framework for understanding the embodied experiences of indirect victims and the interactions between lived experiences and environments through the experience of suffering. I argue this represents an important next step toward opening a dialogue between key actors—indirect victims, their support networks and communities, care providers, government institutions, and scholars from different disciplines. This dialogue, in turn, can contribute to the development of integrative support programs and public policies better aligned with the full complexities of the indirect victims’ lived experiences. I believe that we can draw inspiration from and advance Saunders’ legacy of “total pain” by applying her insights to the multidimensional suffering that attends disappearances, and thereby help alleviate this suffering currently affecting thousands of families in Mexico.

## Data Availability

The original contributions presented in the study are included in the article/supplementary material, further inquiries can be directed to the corresponding author.

## Footnotes

1 According to the International Convention for the Protection of All Persons from Enforced Disappearance, “‘victim’ means the disappeared person and any individual who has suffered harm as the direct result of an enforced disappearance.” (Article 24, Office of the High Commissioner, Human Rights, United Nations (OHCHR), 2026). However, in the literature and the public domain, the *desaparecidos* are usually regarded as the direct victims, their relatives as the indirect or secondary victims.

2 An enforced disappearance is the arrest, detention or abduction of persons by, or with the authorization, support or acquiescence of, a State or a political organization, followed by a refusal to acknowledge that deprivation of freedom or to give information on the fate or whereabouts of those persons, with the intention of removing them from the protection of the law for a prolonged period of time (United Nations, 2006).

3 In 2025, the United Nations Committee on Enforced Disappearances invoked Article 34 in the case of Mexico, based on information indicating the widespread or systematic nature of this phenomenon. On April 2nd, 2026, the committee requested that the General Assembly address this case (Office of the High Commissioner, Human Rights, United Nations (OHCHR), 2026).

4 The original says, Ley general en materia de desaparición forzada de personas, desaparición cometida por particulares y del sistema nacional de búsqueda de personas.

5 The original says Si alguien comete un delito debe estar en prisión, no muerto o desaparecido.

6 The original says: Aunque sea un huesito para darle cristiana sepultura.
